# Post Hoc Subgroup Analysis by Disease Severity in a Non-Inferiority Trial of Donepezil 27.5 mg Transdermal Formulation in Japanese Patients With Mild-to-Moderate Alzheimer Disease

**DOI:** 10.1097/WAD.0000000000000738

**Published:** 2026-07-06

**Authors:** Yu Nakamura, Takuma Tsushio, Masaya Tanahashi, Hideki Suganami, Takumi Omori, Kenichi Nishiyama, Hiroshi Aoki, Naoki Nagakura

**Affiliations:** * Department of Neuropsychiatry, Faculty of Medicine, Kagawa University; ‡ Department of Clinical Development; § Medical Affairs, Teikoku Seiyaku Co., Ltd., Kagawa; † Department of Clinical Data Science, Data Science Center, Kowa Company, Ltd., Chuo-ku, Tokyo, Japan

**Keywords:** Alzheimer disease, cholinesterase inhibitors, donepezil, patches, disease severity, cognition

## Abstract

**Background:**

The efficacy of cholinesterase inhibitors varies depending on the severity of Alzheimer disease. We examined the effects of the disease severity on the efficacy of donepezil 27.5 mg patches in Japanese patients with Alzheimer disease.

**Methods:**

A post hoc analysis of covariance using per-protocol set in a noninferiority study of donepezil 27.5 mg patches with donepezil hydrochloride 5 mg tablets (JapicCTI-194582) was conducted after imputation of missing data using multiple imputations.

**Results:**

No apparent imbalances in the extracted confounding factors were observed between the 2 treatment groups. The baseline value of the Alzheimer Disease Assessment Scale (Japanese version) cognitive subscale was adjusted using analysis of covariance. Least-squares mean of changes from baseline in the Alzheimer Disease Assessment Scale (Japanese version) cognitive subscale at week 24 for each group were −1.661 (−2.681 to −0.640) (donepezil patch) and 0.065 (−0.987 to 1.117) (donepezil hydrochloride tablet). The difference in the least-squares mean (95% CI) between 2 groups was −1.726 (−3.1913 to −0.2602, *P*=0.021).

**Conclusions:**

A post hoc analysis of covariance using the per-protocol set suggested that donepezil patches may be more effective than donepezil tablets in slowing cognitive decline in patients with mild Alzheimer disease.

A hallmark of Alzheimer disease is the reduction of both acetylcholine levels and the activity of its synthesizing enzyme, choline acetyltransferase, in the forebrain. This forms the basis of the “cholinergic hypothesis” of dementia, which identifies cholinergic hypofunction as a key pathologic feature of the disease.^1,2^ Donepezil is widely used to suppress cognitive decline in patients with Alzheimer disease-related dementia.^3^ However, oral formulations are not suitable for patients with dementia who have dysphagia, refuse to take tablets, or struggle with medication adherence due to cognitive decline. Transdermal formulations have become a valuable alternative for patients with dementia. Transdermal patches offer several pharmacokinetic advantages, including a slower initial rise in the plasma drug levels compared with oral formulations. Once steady-state levels are reached, the drug concentration is more easily maintained while the patch is in place. These characteristics are believed to improve patient adherence.

We developed a transdermal formulation of donepezil, a widely used cholinesterase inhibitor, with pharmacokinetic properties comparable to those of donepezil hydrochloride oral tablets, as evaluated by AUC_0__−24 h_.^4,5^ It was approved in Japan for the treatment of cognitive decline in patients with dementia due to Alzheimer disease, based on demonstrating the noninferiority of a 27.5 mg donepezil patch compared with a 5 mg donepezil hydrochloride tablet, as well as its long term safety.^6–8^ A subgroup analysis of the study suggested that baseline Mini-Mental State Examination (MMSE) scores (≥20 and <26) may influence the efficacy of the donepezil patch.^6^ A European multicentre prospective observational study indicated that disease severity affected the efficacy of the donepezil tablet.^9^ Therefore, we examined the suggested findings using a statistical method to correct for confounding factors.

## METHODS

We conducted a post hoc analysis of covariance using the per-protocol set (PPS) for the mild patient group (20≤ MMSE <26), who showed a numerically greater reduction in cognitive decline with the donepezil patch compared with donepezil tablets in a noninferiority study (JapicCTI-194582). The protocol for the original study (JapicCTI-194582) was approved by the Institutional Review Boards of the institutions where the study was conducted and conformed to the provisions of the Declaration of Helsinki. Institutional Review Board approval was obtained before the initiation of this study.

**FIGURE 1:**
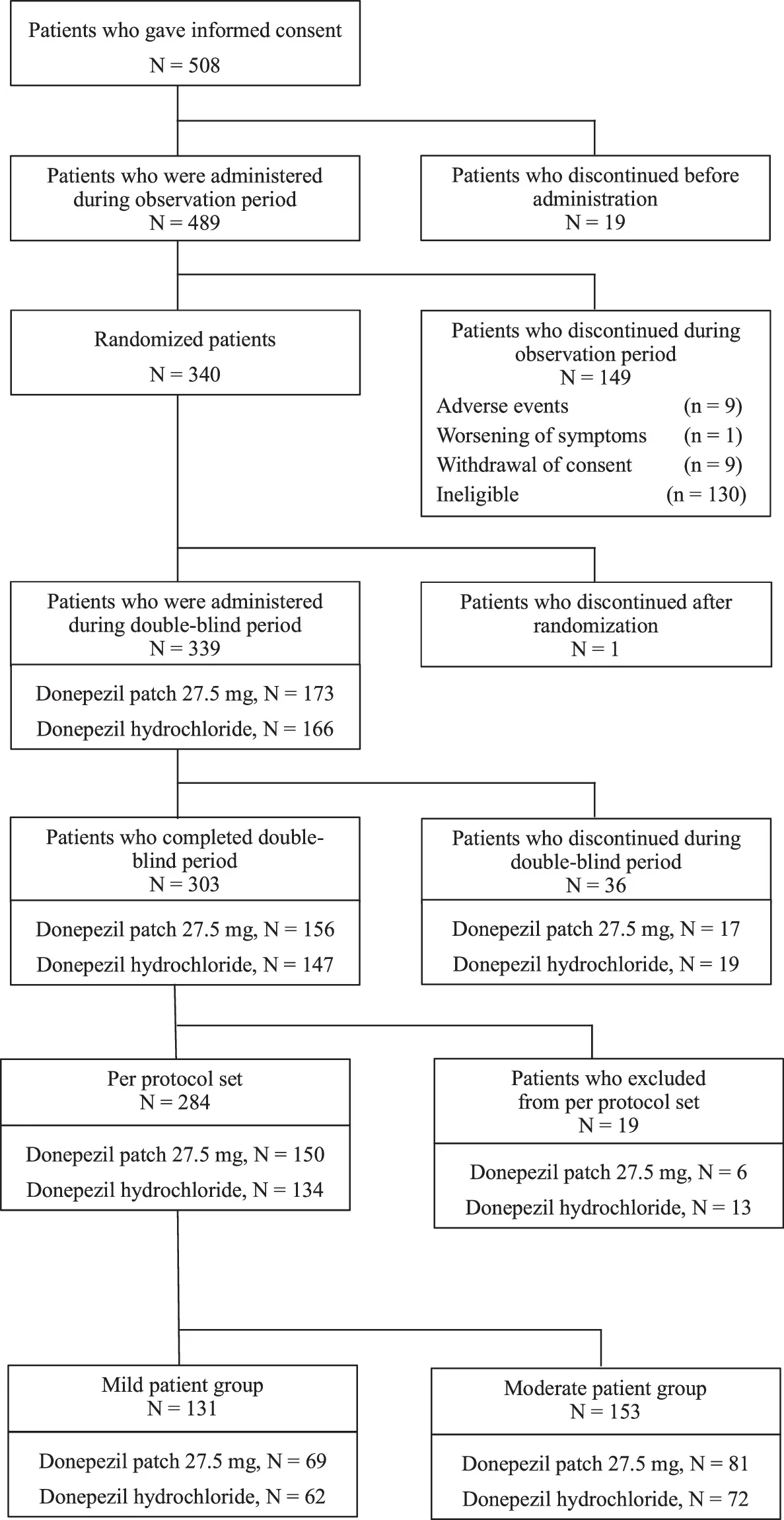
Patient disposition.

The study had 2 phases: a 4-week single-blind observation phase, followed by a 24-week double-blind phase. During the double-blind phase, patients were randomly assigned in equal numbers to either a 27.5 mg donepezil patch group or a 5 mg oral donepezil hydrochloride tablet group (1:1 ratio) using block randomization. Cognitive function was measured at the end of the double-blind phase (24 wk) using the Japanese version of the Alzheimer Disease Assessment Scale (Japanese version) (ADAS-Jcog). Out of 340 randomized patients, 303 completed the double-blind phase, and 284 patients (donepezil patch 27.5 mg, n=150; donepezil hydrochloride, n=134) were included in the PPS (Fig. 1). Noninferiority of the donepezil patch compared with the oral tablet was demonstrated in the PPS using a covariance analysis model. In addition, the patch demonstrated good tolerability, with a safety profile comparable to that of the tablet.

Confounding factors were identified using a directed acyclic graph. Missing data were imputed using multiple imputation (100 times) by group, assuming missingness at random. The baseline ADAS-Jcog value and the ADAS-Jcog values at weeks 4, 12, and 24 were included in the imputation model. These variables were imputed using a linear regression based on the fully conditional specification method. After performing multiple imputations, we conducted a post hoc analysis of covariance on the PPS of the mild group. The baseline ADAS-Jcog value was incorporated as a covariate in the covariance model analysis. The baseline value was used as a covariate to improve the precision of the treatment effect estimate. Multiple results were obtained using the Rubin rule.

## RESULTS

**FIGURE 2:**
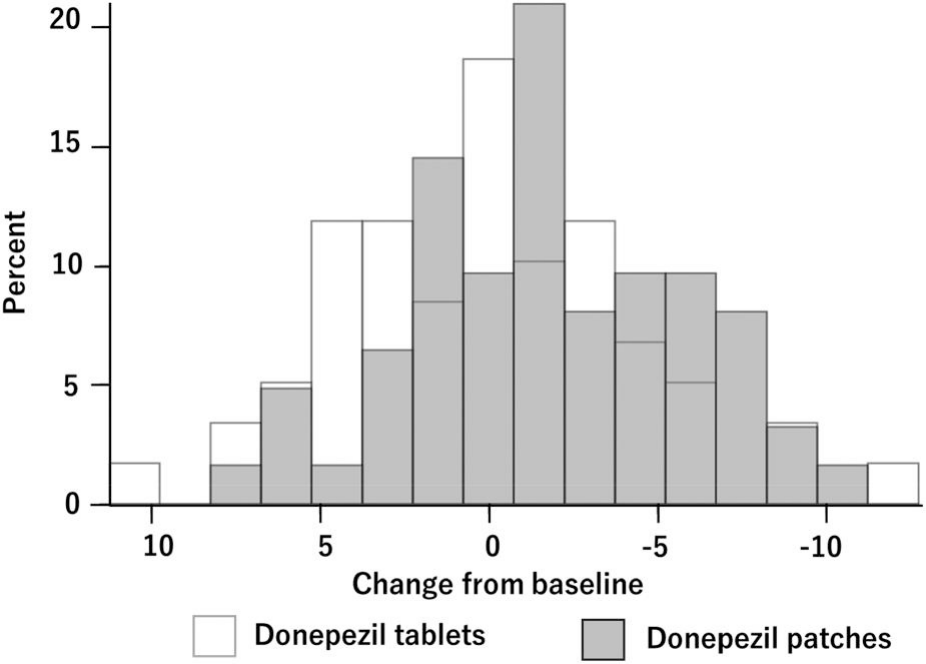
Distribution of change from baseline in ADAS-Jcog scores. ADAS-Jcog indicates Alzheimer Disease Assessment Scale (Japanese version).

**FIGURE 3:**
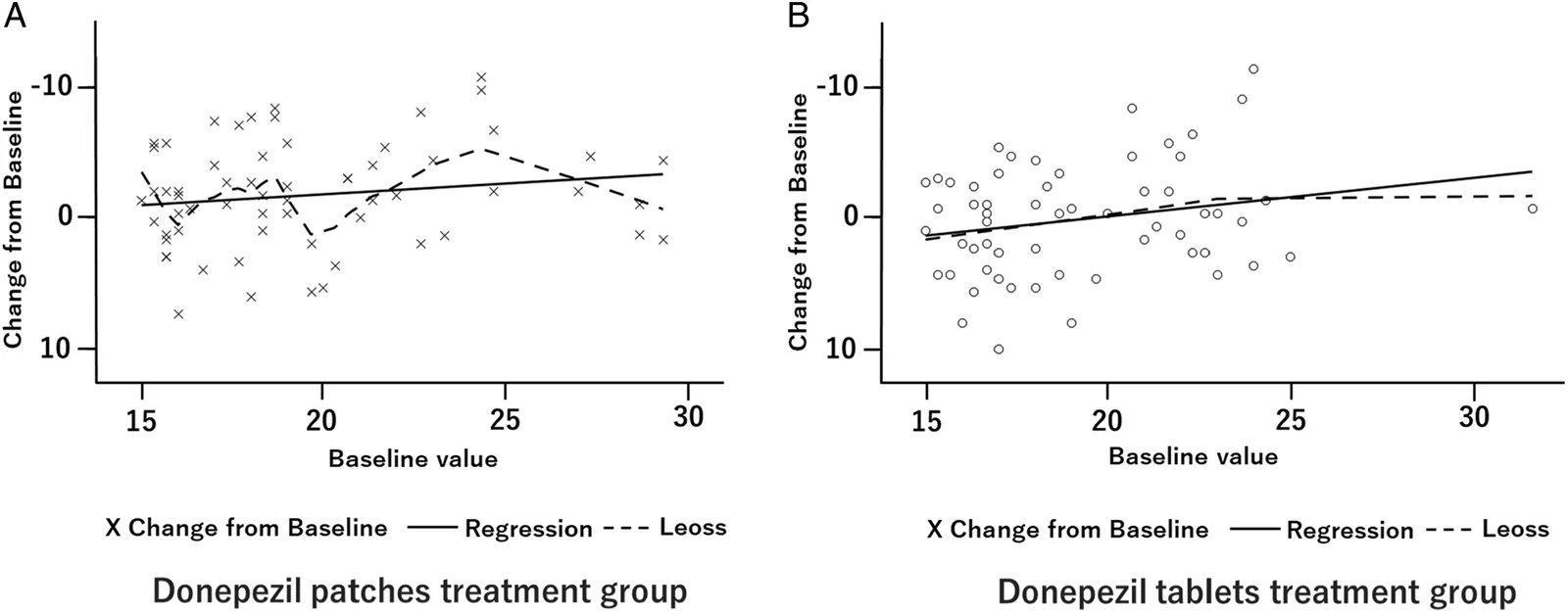
Examination of the relationship between the change from baseline in ADAS-Jcog scores and the baseline value. ADAS-Jcog indicates Alzheimer Disease Assessment Scale (Japanese version).

We confirmed that all necessary assumptions for the analysis of covariance were met. Age, weight, sex, baseline cognitive function, and prior use of cholinesterase inhibitors were identified as the confounding factors affecting cognitive function. No notable imbalances were observed across these factors (Table 1). The distribution of changes from baseline in ADAS-Jcog scores appeared ∼ normal based on the histogram, and no significant violation of homogeneity of variance between groups was detected, as confirmed by both the histogram (Fig. 2) and an *F*-test (*F*-value=1.12, *P*=0.6531). Regression analysis indicated a ∼ linear relationship between baseline ADAS-Jcog scores and changes from baseline (Fig. 3A, B). The homogeneity of the regression slopes between treatment groups was assessed statistically, and no significant differences were observed.

**TABLE 1:** Extracted Confounding Factors on the Analyzed Population

	Donepezil patches	Donepezil tablets
Demographics	n=69	n=62
Age (y)		
Mean (SD)	79.65±5.46	77.74±5.95
Median (Min, Max)	80.00 (66.00, 91.00)	78.00 (61.00, 88.00)
Sex		
Female	46 (66.7)	34 (54.8)
Male	23 (33.3)	28 (45.2)
Weight (kg)		
Mean (SD)	51.69±7.93	54.74±11.40
Median (Min, Max)	51.50 (33.10, 70.80)	54.45 (34.80, 82.20)
MMSE (baseline)		
Mean (SD)	22.30±1.74	22.48±1.78
Median (Min, Max)	23.00 (20.00, 26.00)	22.00 (20.00, 26.00)
ADAS-Jcog total score(baseline)		
Mean (SD)	19.55±3.93	19.16±3.32
Median (Min, Max)	18.33 (15.00, 29.33)	18.17 (15.00, 31.67)
Prior treatment		
No	30 (43.5)	26 (41.9)
Yes	39 (56.5)	36 (58.1)

ADAS-Jcog indicates Alzheimer Disease Assessment Scale (Japanese version); Max, maximum; Min, minimum; MMSE, Mini-Mental State Examination; SD, standard deviation.

Summary statistics for ADAS-Jcog scores and changes from baseline at each visit are presented in Table 2. Analysis of covariance was conducted following multiple imputation, and results were pooled using the Rubin rule. The estimated least-squares mean change from baseline in ADAS-Jcog at week 24 was −1.661 (−2.681 to −0.640) in the patch group and 0.065 (−0.987 to 1.117) in the tablet group (Table 3). The between-group difference in least-squares means was −1.726 (95% CI: −3.1913 to −0.2602), indicating that the patch group showed significantly greater improvement in cognitive function compared with the tablet group in patients with mild Alzheimer disease (*P*=0.021).

**TABLE 2:** The Values and Change in ADAS-Jcog at Each Visit From Baseline

	Donepezil patches	Donepezil tablets
Baseline		
n	69	62
Mean (SD)	19.55±3.93	19.16±3.32
Median (Min, Max)	18.33 (15.00, 29.33)	18.17 (15.00, 31.67)
Week 4		
n	69	59
Mean (SD)	19.72±5.20	19.59±4.50
Median (Min, Max)	20.00 (9.33, 32.33)	19.33 (8.67, 30.33)
Change from baseline		
Mean (SD)	0.17±3.89	0.39±3.90
Median (Min, Max)	−0.33 (−10.00, 9.67)	0.33 (−12.00, 13.33)
Week 12		
n	66	59
Mean (SD)	17.77±4.68	19.67±4.80
Median (Min, Max)	18.00 (8.67, 29.33)	19.67 (9.99, 29.00)
Change form baseline		
Mean (SD)	−1.53±3.72	0.43±4.36
Median (Min, Max)	−1.34 (−11.00, 8.00)	0.34 (−10.67, 11.00)
Week 24		
n	62	59
Mean (SD)	17.88±5.16	19.35±4.78
Median (Min, Max)	17.33 (9.67, 31.00)	19.00 (11.67, 31.00)
Change from baseline		
Mean (SD)	−1.74±4.02	0.10±4.26
Median (Min, Max)	−1.67 (−10.66, 7.33)	−0.33 (−11.33, 10.00)

ADAS-Jcog indicates Alzheimer Disease Assessment Scale (Japanese version); Max, maximum; Min, minimum; SD, standard deviation.

**TABLE 3:** Change in ADAS-Jcog Score From Baseline in Patients With Mild Disease

	Donepezil patches	Donepezil tables
Change		
Number of patients	69	62
Least-squares mean (SE)	−1.661 (0.5206)	0.065 (0.5367)
95% CI	−2.681 to −0.640	−0.987 to 1.117
Between-group difference		
Least-squares mean (SE)	−1.726 (0.7477)*	—
95% CI	−3.1913 to −0.2602	—

* *P*=0.0210.

ADAS-Jcog indicates Alzheimer Disease Assessment Scale (Japanese version); SE, standard error.

## DISCUSSION

The post hoc analysis of covariance using the PPS indicated that donepezil patches were more effective than tablets in ameliorating cognitive decline in patients with mild Alzheimer disease.

As Alzheimer disease progresses, deterioration of the cholinergic system leads to further cognitive decline.^1^ Cholinesterase inhibitors provide symptomatic relief by improving cognitive function; however, their efficacy tends to decrease as the disease advances.^2^ While the same dosage is generally used in mild-to moderate cases, a higher dose is often required in severe cases.^10,11^

A European multicentre prospective observational study reported that donepezil 10 mg tablets provided greater cognitive and functional benefits in patients with mild disease than in those with moderate disease.^9^ However, approved dosages for mild to moderate Alzheimer patients differ between Europe and Japan, 10 mg/d in Europe versus 5 mg/d in Japan, making it difficult to directly apply European findings to the Japanese population.

Stronger inhibition of acetylcholinesterase by donepezil has been correlated with less cognitive deterioration.^12^ The inhibitory effect in the brain is estimated based on plasma donepezil concentrations.^13^ Notably, donepezil patches provide a higher trough concentration than oral tablets (24.58 ng/mL and 21.79 ng/mL, respectively), suggesting more stable acetylcholinesterase inhibition at steady state.^5^ This stable inhibition may explain the superior cognitive outcomes observed with the patch formulation in mild Alzheimer patients.

This study has 2 key limitations. First, the findings were derived from an exploratory post hoc subgroup analysis, with statistical significance observed only in the PPS, not in the full analysis set. Second, there may be imbalances in unmeasured confounding factors (eg, education level or lifestyle habits). Therefore, these results should be interpreted with caution, and confirmation through a well-designed, prospective study is necessary.
